# Prolonged paralysis following mivacurium administration in a pediatric patient with previously undiagnosed pseudocholinesterase deficiency: a case report

**DOI:** 10.1186/s12887-025-05996-9

**Published:** 2025-08-11

**Authors:** Davut Deniz Uzun, Katharina Wildenberg, Fabian Ruping, Jens H. Westhoff, Felix C.F. Schmitt, Markus A. Weigand, Aleksandar R. Zivkovic

**Affiliations:** 1 Department of Anesthesiology, Medical Faculty Heidelberg, Heidelberg University, Heidelberg, Germany; 2 Division of Pediatric Surgery, Department of General, Visceral and Transplantation Surgery, Medical Faculty Heidelberg, Heidelberg University, Heidelberg, Germany; 3 Department I, Center for Pediatric and Adolescent Medicine, Medical Faculty Heidelberg, Heidelberg University, Heidelberg, Germany

**Keywords:** Mivacurium, Pseudocholinesterase deficiency, Case report, Prolonged neuromuscular block, Pediatric anesthesia

## Abstract

**Background:**

Pseudocholinesterase, an enzyme produced by the liver and abundantly present in plasma, plays a role in the metabolism of neuromuscular blocking agents such as mivacurium. The administration of mivacurium to patients with pseudocholinesterase deficiency during general anesthesia has been associated with the occurrence of prolonged postoperative paralysis, a rare but potentially severe side effect.

**Case presentation:**

A 24-month-old girl underwent routine, elective minor surgery. The neuromuscular blocking agent mivacurium was used during general anesthesia. Following the procedure, there was no spontaneous recovery of breathing. The peripheral nerve stimulator used to measure neuromuscular relaxation did not elicit any responses. The patient was subsequently transferred to the pediatric intensive care unit, where she was successfully weaned from the ventilator and extubated four hours later. The following day, the child was deemed fit for discharge from the clinic, exhibiting no residual symptoms. A targeted laboratory analysis conducted subsequent to the event revealed a cholinesterase level of 4176 U/l (normal values 4260–12920 U/l), a cholinesterase dibucaine-number of 12 (normal values > 70) and cholinesterase fluoride-number of 29 (normal values 55–60).

**Conclusions:**

This is a case report of pseudocholinesterase deficiency in a 24-month-old child undergoing minor surgery. Quantitative neuromuscular monitoring should be used routinely to recognize prolonged muscle relaxation. If the diagnosis of pseudocholinesterase deficiency is confirmed, the patient should be given information about the disorder, the associated risks, inheritance and the need for testing in other family members.

## Background

Butyrylcholinesterase, also referred to as plasma cholinesterase, serum cholinesterase, or pseudocholinesterase (PChE), is an enzyme that is synthesized in the liver and is abundantly present in plasma and in the majority of human tissues. The physiological function of PChE remains unclear. However, it exhibits similar protective properties to acetylcholinesterase, acting as a safeguard against acetylcholinesterase inhibitors and inhaled toxins [1, 2]. PChE deficiency is a rare enzymatic disorder with significant implications for pediatric anesthesia, particularly due to the prolonged effects of mivacurium [3, 4]. PChE deficiency is the result of mutations in the *butyrylcholinesterase* (BCHE) gene, which encodes the PChE enzyme responsible for metabolizing choline esters [4]. Patients with PChE deficiency may experience prolonged muscle paralysis and apnea following the administration of neuromuscular blocking agents, such as mivacurium and succinylcholine [5]. The aforementioned factors can present challenges during the recovery of spontaneous breathing, thus increasing the risk of respiratory complications [6]. PChE deficiency can manifest as either an acquired or congenital disorder. The latter is inherited in an autosomal recessive pattern [7]. In children with congenital PChE deficiency, the condition may remain asymptomatic until exposure to triggering agents, at which point the deficiency is often revealed. This is typically observed under the controlled environment of general anesthesia. The incidence of the deficiency varies among populations. Studies have demonstrated a prevalence ranging from 1 in 3,000 (homozygous) to 1 in 5,000 in Caucasian populations. However, the condition has been less extensively studied in pediatric cohorts [8]. Given that children with undiagnosed PChE deficiency may encounter unforeseen difficulties during anesthesia, it is imperative for anesthetists and pediatricians to comprehend the genetic and biochemical factors associated with PChE deficiency. The routine implementation of quantitative neuromuscular monitoring has been demonstrated to facilitate the identification of cases involving the unrecognised prolonged action of neuromuscular blocking agents. Consequently, the extubation practices in some hospitals, which are not accompanied by quantitative neuromuscular monitoring, should be re-evaluated. The following case report details the case of a child who experienced an unusually prolonged apnea and required ventilation for a period of four hours in the pediatric intensive care unit.

## Case presentation

The child (age: 24 months; height: 90 cm, weight: 11 kg, BMI 13.58 kg/m²) was referred to the pediatric surgical center of the university hospital in Heidelberg, Germany by colleagues in the dermatology department for the routine removal of two skin lesions with the risk of malignancy. The preoperative anesthetic examination demonstrated that the child’s development was aligned with their age-appropriate milestones and did not indicate the presence of any systemic diseases. The preoperative vital signs and laboratory findings were within the normal range. The patient had no documented history of medication use, and neither she nor any other family member had a history of anesthetic complications associated with the muscle relaxants succinylcholine or mivacurium. The preoperative physical examination conducted upon admission yielded results within the normal range.

**Fig. 1:**
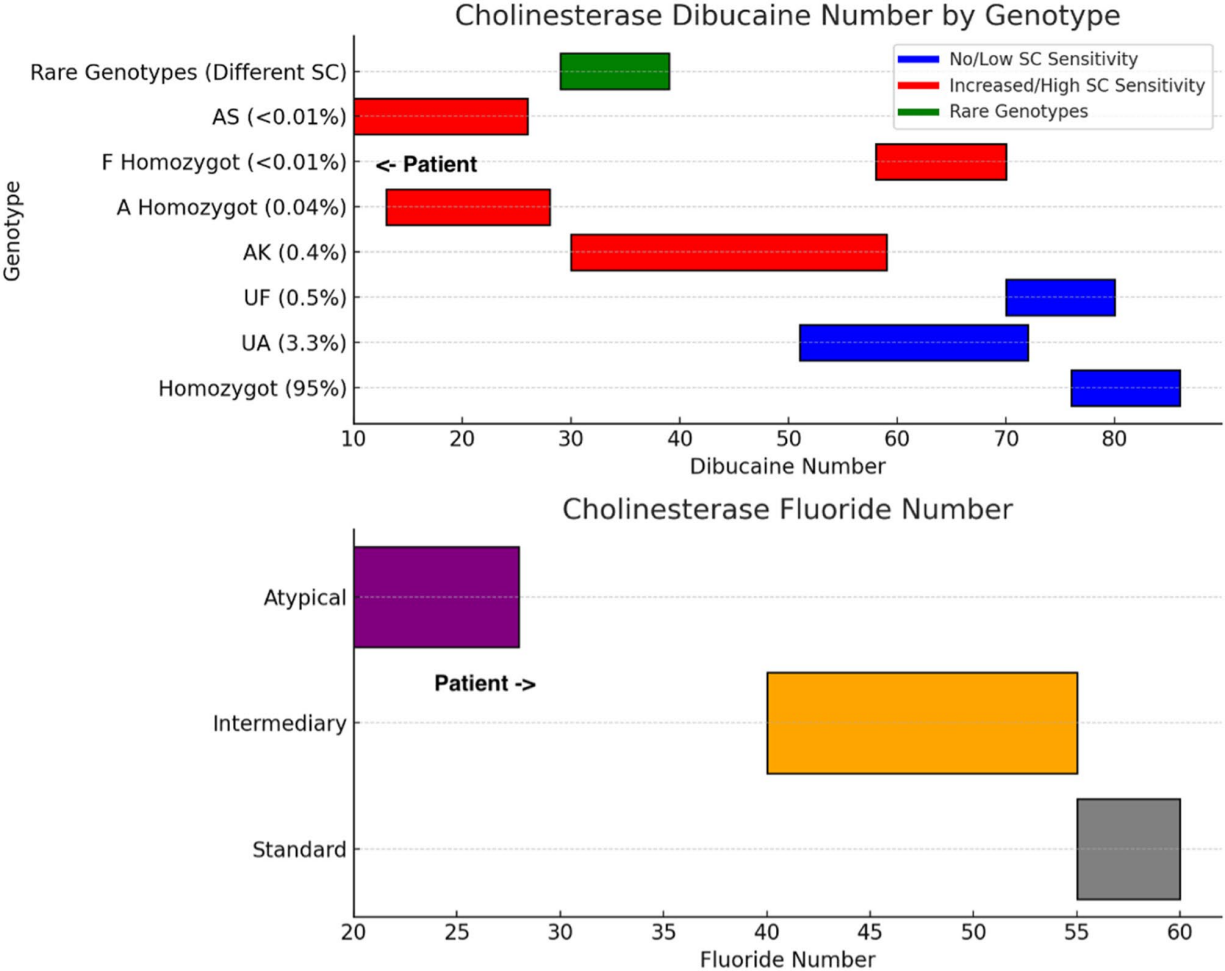
Overview of Succinylcholine Sensitivity (SC) Across Genotypes: Cholinesterase Dibucaine and Fluoride Numbers. Top chart shows ranges of cholinesterase dibucaine number for various genotypes: Blue: Genotypes with no or low SC sensitivity; Red: Genotypes with increased or high SC sensitivity; Green: Rare genotypes with diverse SC sensitivities. Bottom chart represents cholinesterase fluoride number standard, intermediary, and atypical ranges, color-coded for clarity: Gray: Standard range; Orange: Intermediary range; Purple: Atypical range

In accordance with the clinic’s internal guidelines, the patient was given premedication with 0.5 mg/kg body weight of midazolam orally before surgery. Venous access was successfully established, and standard anesthesia monitoring and preoxygenation were carried out in accordance with current international standards. General anesthesia was induced with propofol at a dose of 5 mg/kg body weight and alfentanil at a dose of 20 µg/kg body weight. A total dose of 2 mg of mivacurium was administered intravenously for the purpose of achieving neuromuscular block. Following a three-minute interval, the oral tracheal intubation was completed without incident, resulting in a “first-pass success”. Anesthesia maintenance was achieved with sevoflurane by inhalation at an age-adapted minimal alveolar concentration (MAC) between 0.8 and 1. Monitoring consisted of non-invasive arterial blood pressure, electrocardiogram (ECG), pulse oximetry, and continuous end-tidal carbon dioxide measurement. The surgery lasted a total of 20 min, after which the child did not exhibit any signs of awakening following the discontinuation of sevoflurane. To identify potential medication errors, a comprehensive review of all medications was conducted, and no evidence of an overdose was found. Consequently, the child was observed to be apneic and unresponsive. The patient exhibited clear clinical signs of stress, including elevated blood pressure, tachycardia, and profuse sweating. A peripheral nerve stimulator did not trigger any neuromuscular responses. Quantitative neuromuscular monitoring was not used in this case. The patient was able to breathe with adequate frequency under ventilator-assisted mode and full pressure support, which failed each time spontaneous breathing trial was conducted. This strongly suggests the effects of a muscle relaxant. No evidence of hypoxemia or other causes were identified at any point during the observation period, as measured by peripheral arterial saturation levels. Consequently, sedation with propofol was reintroduced and maintained at a dosage of 4 mg/kg/hr. We hypothesized a possible muscle relaxant overdose or an inability of the metabolism thereof, and therefore decided to transfer the sedated, intubated and ventilated patient to the pediatric intensive care unit (PICU). A re-assessment was conducted in the PICU, with all potential differential diagnoses taken into account. A hematological examination, routine whole blood cell counts, coagulation studies, electrolyte estimates, and pseudocholinesterase levels were sent to a specialized laboratory for further analysis. Subsequently, the child was transferred to a spontaneous ventilation mode and, following the clinical full recovery of the musculoskeletal system, was extubated without incident. Consequently, the child regained consciousness approximately four hours after the surgical procedure. The subsequent course of hospitalization was unremarkable, and the child made a full recovery without short-term complications. The child was discharged the following day in a state of optimal health. A few days after the child was discharged, the results of the specialized laboratory tests arrived. Table 1; Fig. 1 show the detailed values and genotypes:

**Table 1:** Results of the laboratory analysis revealing pseudocholinesterase deficiency

Laboratory blood analysis	Patient values	Normal values
Cholinesterase	4176 U/l	4260–12,920 U/l
Cholinesterase Dibucaine-Number	12	> 70
Cholinesterase Fluoride-Number	29	55–60

## Discussion and conclusions

We present a case of prolonged muscle relaxation after a single dose of mivacurium in a 24-month-old child. Even though the problems associated with succinylcholine and mivacurium have been known for decades, their incidence in pediatric anesthesia is very rare. Although persistent neuromuscular block due to PChE deficiency is a rare side effect of administration of mivacurium or succinylcholine, it could result in a condition that is clinically challenging to diagnose and can be potentially lethal.

Patients with PChE deficiency exhibit a high degree of sensitivity to mivacurium and succinylcholine. Consequently, prolonged post-anesthetic apnea can occur, which may ultimately lead to death after administration of these drugs [9]. The results of a recent study indicate that a genetically determined PChE deficiency is a significant contributing factor in the occurrence of prolonged neuromuscular block following mivacurium administration. In fact, the study revealed that PChE deficiency was present in 80% of patients who experienced an adverse event [10]. The genetics of PChE were first described by Kallow and Genest and are widely regarded as the foundation of pharmacogenetics/pharmacogenomics research. At present, in excess of 60 genetic variants are known [11]. It is postulated that genetically inherited deficits are autosomal and recessive in character and can occur as a result of mutations [4]. Additionally, numerous non-genetic factors can contribute to diminished PChE activity, including malnutrition, renal disease, hepatic disease, neoplastic processes, thermal injury, hypothyroidism and burns [12]. Additionally, certain medications, including oral contraceptives, clindamycin, insecticides, and metoclopramide, have been linked to PChE deficiency [13, 14]. PChE deficiency is frequently not identified until after general anesthesia has been administered, when breathing is paralyzed for an extended period of time following the administration of mivacurium. There is no causal treatment for PChE deficiency, but some approaches are available, for example, the administration of human cholinesterase, neostigmine or fresh frozen plasma, however, these therapies can also be associated with possible side effects and complications [15]. Therefore, from the authors’ point of view, the primary goal of treatment is to provide ventilation and maintenance of sedation/amnesia until the drug has been cleared from the neurosynaptic cleft and neuromuscular function has been restored.

Furthermore, reduced PChE activity has also been described in critically ill patients [16]. A study revealed a reduction in PChE activity in patients with systemic inflammation compared to healthy individuals. Despite previous assumptions that liver function was essential for PChE production, studies have shown that the reduced PChE activity associated with systemic inflammation occurs independently of liver function in these patients [16]. The current practice in some hospitals of extubating patients without quantitative neuromuscular monitoring should be critically questioned. The lack of neuromuscular monitoring is certainly also the major limitation in this case report. One of the most important learning goals of this case report is that quantitative neuromuscular monitoring should be performed in every patient after the administration of NMBAs, even after a single dose. This can be achieved using a train-of-four measurement (AMG or EMG), for example, and the patient should only be extubated if the TOF ratio is at least 0.9. If residual paralysis is present, it is recommended the reversal of NMBAs prior to tracheal extubation. This approach was associated with a significant reduction in postoperative pulmonary complications [17]. Therefore, it is of primary importance to ensure a TOF ratio greater than 0.9 prior to extubation. The method of reversal (neostigmine, sugammadex or spontaneous recovery) is therefore secondary. Nevertheless, in view of sugammadex, it is important to reconsider the frequent use of mivacurium/succinylcholine. On the other hand, however, the side effects of sugammadex should also be taken into account in the decision [18].

In this case, the patient’s PChE deficiency was evident despite the fact that the conventionally measured cholinesterase activity showed only a mild reduction, which typically does not lead to any noticeable clinical effects. This observation highlights that a preoperative screening protocol based solely on conventional cholinesterase measurements would not have reliably predicted the risk of prolonged paralysis, as such slight reductions are generally not considered clinically impactful. Consequently, implementing a widespread preoperative screening protocol specifically for pseudocholinesterase deficiency in pediatric patients would likely be inefficient and resource-intensive unless there were specific reasons or risk factors indicating a higher likelihood of this deficiency.

The rate of intraoperative awareness in pediatric anesthesia seems to be higher than in adult anesthesia [19]. Undetected prolonged neuromuscular block can also lead to intraoperative awareness. Being conscious during surgery is a traumatic event that can lead to chronic post-traumatic stress disorder [20]. Therefore, every effort should be made to prevent intraoperative awareness. A three-month follow-up was conducted to evaluate the potential development of post-traumatic stress disorder or other psychosocial sequelae of the brief awareness episode. The results indicated the absence of any abnormalities. The growing preference for outpatient treatment in European medicine will, in the future, result in an increase in the number of minor surgical procedures being performed on an outpatient basis. In the context of postoperative care, anesthesiologic monitoring of patients assumes particular relevance. It is not uncommon for day clinics to lack the necessary resources to provide prolonged postoperative monitoring and, if required, ventilation. This is undoubtedly attributable to deficiencies in infrastructure and staffing. In conclusion, it can be stated that all medical professionals involved in the treatment of pediatric patients requiring general anesthesia are informed about the potential adverse effects of anesthesia and also discuss the possibility of postoperative ventilation with the families.

### Conclusion for practice

- The residual neuromuscular block after mivacurium/succinylcholine is a well-known effect in patients with pseudocholinesterase deficiency.
- If not recognized by the anesthesiologist, this may cause complications such as awareness and residual paralysis leading to hypoxia.
- To prevent this, the anesthesiologist should perform quantitative neuromuscular monitoring, for example through a train-of-four measurement on every patient who has received neuromuscular blocking agents before tracheal extubation.
- A pseudocholinesterase deficiency may remain asymptomatic for decades, often only being identified when patients undergo anesthesia and receive a neuromuscular blocking agent that depends on pseudocholinesterase for its metabolism.

## Data Availability

No datasets were generated or analysed during the current study.

## Abbreviations

BCHE: Butyrylcholinesterase
BMI: Body-mass-index
FFP: Ffresh-frozen plasma
MAC: Mminimal alveolar concentration
NMBA: Neuromuscular blocking agents
PChE: Pseudocholinesterase
PICU: Pediatric intensive care unit
